# The Efficacy of Cancer Immunotherapies Is Compromised by *Helicobacter pylori* Infection

**DOI:** 10.3389/fimmu.2022.899161

**Published:** 2022-05-23

**Authors:** Paul Oster, Laurie Vaillant, Brynn McMillan, Dominique Velin

**Affiliations:** Service of Gastroenterology and Hepatology, Centre Hospitalier Universitaire Vaudois, University of Lausanne, Lausanne, Switzerland

**Keywords:** *Helicobacter pylori*, cancer, immunotherapy; personalized medicine, gut microbiota, immune checkpoint inhibitors

## Abstract

*Helicobacter pylori* infects the gastric mucosa of a large number of humans. Although asymptomatic in the vast majority of cases, *H pylori* infection can lead to the development of peptic ulcers gastric adenocarcinoma and mucosa-associated lymphoid tissue (MALT) lymphoma. Using a variety of mechanisms, *H pylori* locally suppresses the function of the host immune system to establish chronic infection. Systemic immunomodulation has been observed in both clinical and pre-clinical studies, which have demonstrated that *H pylori* infection is associated with reduced incidence of inflammatory diseases, such as asthma and Crohn’s disease. The introduction of immunotherapies in the arsenal of anti-cancer drugs has revealed a new facet of *H pylori*-induced immune suppression. In this review, we will describe the intimate interactions between *H pylori* and its host, and formulate hypothtyeses describing the detrimental impact of *H pylori* infection on the efficacy of cancer immunotherapies.

## Introduction

Antitumor immunity is a critical concept in oncology that refers to the ability of the immune system to prevent the formation, growth and metastases of tumors (1). Following the recognition of the cellular and genetic alterations that characterize cancer cells, the immune system is capable of generating effector T cell responses that function to eliminate neoplastic cells (2). The generation of tumor-specific immune responses begins when antigen presenting cells (APCs), such as dendritic cells (DCs), capture tumor-specific antigens that have been released by cancer cells and migrate to secondary lymphoid structure(s) which drain the tumor site (2). The processed antigen is then cross-presented by DCs most commonly *via* major histocompatibility complex (MHC) class I molecules to CD8^+^ T cells, allowing for their activation, differential expansion and subsequent migration to the tumor site (2, 3). Once at the tumor site, activated CD8^+^ T cells recognize the tumor-specific antigen present at the surface of the cancer cell through the interaction between the antigenic peptide-MHC class I complex and the T cell receptor (TCR) (2). This interaction induces the release of highly cytotoxic molecules from CD8^+^ T cells including perforin, granzyme, tumor necrosis factor (TNF) α and interferon (IFN) γ, which leads to the induction of cancer cell death (4). A number of individualistic factors such as tumor and germline genetics, age, and the presence of infectious agents may impact the efficacy of this antitumor response (2). Several additional immune cell populations are implicated in antitumor immunity including CD4^+^ T cells, natural killer cells (NK cells), T regulatory cells (Treg cells), B cells, various DC subsets, macrophages, monocytes and myeloid-derived suppressor cells (MDSCs).

Cancer immunotherapy is a form of cancer treatment that aims to increase antitumor immunity (5). Currently, there exists six major classes of immunotherapies including lymphocyte promoting cytokines, chimeric antigen receptor (CAR-T) T cells, T cell receptor-engineered T cell (TCR-T), costimulatory receptor agonists, anticancer vaccines and immune checkpoint inhibitors (6, 7). Immune checkpoint receptors, including Cytotoxic T-lymphocyte-associated protein 4 (CTLA-4) and Programmed cell death 1(PD-1)/Programmed cell death-ligand 1 (PD-L1), are coinhibitory receptors that function in the attenuation of immune cell activation and have recently become a main target in the treatment of cancer (8). Increasing the amount of cytotoxic tumor-infiltrating lymphocytes using vaccination strategies as well as blocking the natural function of the CTLA4 and/or PD-1/PD-L1 immune checkpoints are attractive approaches for anticancer therapies as they enable the host to mount an appropriate immune response to counteract tumor formation, growth and metastasis. Presently, there exist two main cancer immune checkpoint blockade therapies that are approved for clinical use for a number of tumor types, including anti-CTLA4 (ipilimumab) and anti-PD-1/PD-L1 (pembrolizumab, nivolumab/atezolizumab), each of which blocks the attenuation of T cell activity, thereby inducing the expansion of CD8^+^ T cells and differentially affecting CD4^+^ T cells (9). Importantly, only a subset of patients respond to these therapies, owing to individualistic factors affecting anticancer immunity as previously described, with additional factors such as the composition of the gut microbiota (2).

### Microbiota: A Key Component Influencing the Efficacy of Cancer Immunotherapy

The microbiota represents a highly complex symbiotic relationship between the human body and microorganisms including bacteria, archaea, eukarya and fungi (10, 11). The gut microbiota, referring to the microbial communities along the gastrointestinal tract, functions fundamentally in the maintenance of metabolic homeostasis alongside protecting the body against pathogens (10). Previous studies strongly suggest that the presence of the gut microbiota is in fact necessary in conferring the anticancer effects elicited by anti-CTLA4 immunotherapy (12). Using mice subcutaneously inoculated with cancer cell lines, Vetizou and colleagues demonstrated that mice housed in specific pathogen-free conditions with fully intact microbiotas, had markedly improved tumor control relative to mice housed in germ free conditions, which lack a functional microbiota, following the administration of anti-CTLA4 immunotherapy (12). More particularly, Vetizou et al. demonstrated that the antitumor effects elicited by CTLA4 blockade in mice were dependent on the presence of *Burkholderiales Bacteroides fragilis* and *Bacteroides thetaiotaomicron* in the gut microbiota (12). Additional bacteria such as *Faecalibacterium prausnitzii, Akkermansia muciniphila*, and *Bifidobacterium* species were also shown to potentiate the efficacy of immune checkpoint inhibitors (13, 14). Importantly, clinical data clearly demonstrate that the intestinal microbiome modulates the anti-cancer immune response (13, 14) and that antibiotics compromise the efficacy of PD-1 blockade therapy (15). Finally, it was recently described that fecal microbiota transplants overcome the resistance to anti–PD-1 therapy in 30% of melanoma patients (16, 17). Collectively, pre-clinical and clinical data demonstrate that the gut microbiota plays an important role in enhancing the anti-cancer immune response following cancer immunotherapies and therefore, is a major research focus for the development of precision medicine and for the improvement of immunotherapy effectiveness.

As described above, some bacterial species can enhance the effectiveness of cancer immunotherapy, whereas other bacterial species have the opposite effect (18). We recently demonstrated that *Helicobacter pylori* is one of the bacterial species which decreases the efficacy of cancer immunotherapies (19). In this review, we describe *Helicobacter pylori* and its host interactions, and discuss the different hypotheses explaining the decrease in efficacy of cancer immunotherapies in *H pylori infected* hosts.

### Helicobacter pylori

*H pylori* are gram-negative microaerophilic bacteria that are largely successful in infecting the stomach mucosa of up to 50% of humans (20). Most commonly colonizing during childhood and persisting throughout life, *H pylori* has been described to have coevolved with humans for nearly 60,000 years (21). The majority of infected individuals remain asymptomatic, suggesting that *H pylori* has become well adapted to the acidic environment of the gastric epithelium (20, 21). *H pylori* infection can be detected by stool antigen test, serology, urea breath test and/or *via* gastric biopsy (22). *H pylori* infection takes place mainly in the family circle and is transmitted through ingesting saliva, vomit or fecal matter (23). Upon entering the stomach, the flagella and spiral shape of the bacterium allows for the penetration of the mucus gastric layer (24). Because a minute number of bacteria traverse the gastric epithelial barrier, *H pylori* is considered a non-invasive bacteria. Most *H pylori* are living in the mucus, but some organisms adhere to the gastric epithelial cells (25) and even have been detected inside the cells (26). Humans are infected by 10^4^ to 10^7^ *H pylori* colony forming units (CFU) per gram of gastric mucus (27). Urease and α-carbonic anhydrase of *H pylori* generate ammonia and HCO_3_^2-^, which increase the low pH of the gastric juice (28, 29). Outer membrane proteins, such as blood group antigen binding adhesin A (BabA), sialic acid-binding adherence (SabA), adherence-associated lipoprotein A/B (AlpA & AlpB) and *H pylori* outer membrane protein Z (HopZ) mediate attachment to gastric epithelial cells (30). *H pylori* effector molecules, such as vacuolating cytotoxin (VacA), cytotoxin- associated gene A (CagA) and components of the type IV secretion system (CagL) modulate the function of gastric epithelial cells, leading to the release of chemokines and nutrients and to the modification of acid secretion (31, 32).

### *H pylori* Infection May Lead to the Development of Gastritis, Peptic Ulcers and Gastric Cancers

*H pylori* infection can be asymptomatic or symptomatic and may conduct to the development of gastritis, peptic ulcers and gastric cancer (20) (**Table 1**). Colonization by *H pylori* induces an inflammatory response, predominantly of the Th1 type. If the infection is not eradicated, chronic gastritis can be life-long (33). Nevertheless, the vast majority of *H pylori*-positive subjects do not suffer from any symptoms and are unaware of the presence of this inflammation in their stomach. The chronic gastritis generates reactive oxygen species that can give rise to DNA damage and consequently initiate the cancer cascade (chronic gastritis-gastric atrophy-intestinal metaplasia-dysplasia-gastric cancer) (34). The tumorigenic role of *H pylori* is further supported by data showing that eradication of *H pylori* reduces the risk of developing *H pylori*–driven gastric cancer (35, 36). However, depending of the stage of the *H pylori*-induced diseases, patients might not benefit from the *H pylori* treatment on the risk to develop gastric cancer (37).

**Table 1:** *Helicobacterpylori* infection promotes or reduces human pathologies.

Pathologies caused by *Helicobacter pylori* infection	
	Gastritis
	Duodenal and gastric ulcers
	Gastric adenocarcinoma
	Gastric MALT lymphoma
Pathologies potentially reduced by *Helicobacter pylori* infection	
	Gastro-esophageal reflux disease
	Esophagitis
	Asthma
	Eosinophilic esophagitis
	Inflammatory bowel disease
	Food allergies

Peptic ulcers are acid-induced lesions found in the stomach and duodenum, characterized by denuded mucosa (38). Ulcers develop at sites where gastric inflammation is most severe and a variety of host and bacterial factors govern the severity of ulcerations (39).

Chronic inflammation can lead to the destruction of gastric glands and the development of fibrosis and intestinal metaplasia (34). Half of the *H pylori*-colonized population develops atrophic gastritis and intestinal metaplasia (34). Patients with low acid output show a rapid progression towards atrophy. Moreover, the multifocal extension of intestinal metaplasia and gastric atrophy will increase the risk of gastric cancer development (34). The development of gastric atrophy or cancer is modulated by host and bacterial factors that cause chronic inflammation, endoplasmic reticulum stress, autophagy, and oxidative stress in the gastric epithelium (40). The risk is increased in *CagA/VacA*-positive strains infected patients (40–42), and also in those with high IL-1 production in response to *H pylori* infection (40, 43). The *VacA* gene is present in most all *H pylori* strains. However, depending on the strain, the toxic activity of VacA may differ. The identified epithelial cells VacA receptors include receptor protein tyrosine phosphatases α (RPTPα) and β (RPTPβ), and low‐density lipoprotein receptor‐related protein‐1 (LRP1) (44). The Type IV secretion system (T4SS) delivers CagA into epithelial cells. This pilus-like structure interacts with β1 integrin at the surface of epithelial cells (30). Injected CagA hijacks multiple intra-cellular signal transduction cascades modulating key pathways involved in the control of apoptosis and self-renewal of the gastric epithelium (45).

Lymphoid tissue is not normally present in the gastric mucosa, but in response to *H pylori* colonization mucosal associated lymphoid tissue (MALT) nearly always develops (46). Rarely, B lymphoma may develop in those MALT structures. Remarkably, following *H pylori* eradication a complete remission is observed in approximately 80% of these patients (47).

### *H pylori* Infection Protects From Gastro-Esophageal Reflux Disease or Esophagitis

Only a very small proportion of patients infected with *H pylori* develop pathologies as described above and paradoxically, studies associate the decrease in the rate of *H pylori* infection with an increased incidence of several diseases (**Table 2**). Indeed, *H pylori* eradication was reported to be associated with gastro-esophageal reflux disease (GERD) or esophagitis (48). The chronic exposure to acid secretion from the stomach of the esophageal mucosa induces GERD. It is hypothesized that the *H pylori*-induced inflammation in the gastric corpus decreases the acid output and prevents patients from contracting GERD (49). Another detrimental association with *H pylori* eradication is weight gain (50). Neuroendocrine cells in the gastric epithelium produce hormones that regulate body weight. Among them, Ghrelin promotes weight gain by decreasing energy consumption whilst leptin increases energy consumption. *H pylori* infection is associated with an increase in plasma leptin levels and a decrease in Ghrelin levels (51). It is believed that the inflammation induced by *H pylori* colonization interferes with those neuroendocrine cells by altering the epithelial cell turnover (51).

**Table 2:** *Helicobacter pylori* infection reduces the efficacy of cancer immunotherapies.

*Helicobacter pylori* infected mice, compared to non-infected, displayed	
	Low response to anti-CTLA-4 therapy
	Low response to anti-CTLA-4/anti-PD-L1 combo therapy
	Low response to cancer vaccination
	Low vaccine-induced inflammatory cytokine production
	Reduced vaccine-induced tumor-specific CD8+ T cell responses
	Decreased dendritic cells cross-presentation activities
*Helicobacter pylori* seropositive non-small cell lung cancer patients, compared to *Helicobacter pylori* seronegative non-small cell lung cancer patients, displayed	
	Low response to anti-PD-1 immunotherapy
	Reduce anti-PD-1-induced tumor expression of type I interferon, IFNγ and IL-6

### *H pylori* Infection Protects From Systemic Inflammatory Diseases

A recently well described positive side effect of *H pylori* infection is protection against asthma and eosinophilic esophagitis (EoE). Asthma is an immune disease in which allergens trigger a Th2-dependent immune response in the lungs, leading to massive infiltration of eosinophils and mast cells, hyper production of mucus and airway obstruction (52–54). EoE is also an immune disease characterized by esophageal dysfunction, eosinophilic inflammation, a thickened mucosa, muscle layer hyperplasia and collagen deposition (55, 56). Impaired epithelial barrier function and food and aeroallergen hypersensitivity trigger EoE symptoms (57). Incidence of asthma and EoE is growing in developed countries and is commonly explained by the hygiene hypothesis (58, 59). This hypothesis is based on the increased hygiene standard and urbanization that is associated with modern lifestyle. Under these conditions, it is thought that humans are exposed to less pathogens or commensals, especially at a young age, leading to the development of autoimmune/inflammatory diseases later in life (60). Several epidemiological studies in the past decade have inversely correlated asthma, multiple sclerosis and EoE with *H pylori* infection (61–64). A potential mechanism of protection against asthma came from a recent study demonstrating that *H pylori* infection, along with VacA and γ-glutamyl transpeptidase (γGT) activity, is able to interfere with dendritic cell (DC) maturation. These immature DCs fail to induce a proinflammatory response but promote Treg cell differentiation. Both immature DCs and Treg cells were shown to be able to migrate to the lungs and exhibit their tolerogenic phenotype, inducing a reduction in immune activation and development of asthma (65). Moreover, immunoregulatory activity of *H pylori* infection has been shown also in the context of inflammatory bowel diseases (IBD). *H pylori* DNA was able to reduce colitis induced by Dextran Sulfate Sodium (DSS) in mice (66). NOD-like receptor pyrin domain-containing-3 (NLRP3)-dependent IL-18 production induced by *H pylori* has been shown to be critical in the reduction of this colitis severity, by *H pylori* infection in this DSS model (67). Cross-sectional studies also showed a positive correlation between *H pylori* loss at the population level in developed countries with increased incidence of IBD (68).

## *H pylori* Infection Decreases the Efficacy of Immune Checkpoint Inhibitors

We recently hypothesized that *H pylori* infection may decrease the effectiveness of ICI therapies. This hypothesis was based on the observation that gut microbiota has been identified as a key driver of ICI efficacy and that as described above *H pylori* actively suppresses the functioning of the immune system (20). Indeed, *H pylori* displays major immunomodulators such as CagA, Lipopolysaccharide (LPS), flagelin, VacA, γGGT and urease among others which differentially impact a number of immune cells in the gastric mucosa as well as systemically (20, 69). Noteworthy, *H pylori* has been shown to modulate key immune cells involved in the generation of the anti-tumor responses. Indeed, *H pylori* was shown to decrease the activities of Th1 and Th17 cells, DCs, macrophages and Natural killer T cells (NKT cells) and to promote the activities of MDSC and Treg cells, suggesting that *H pylori* infected hosts might be less responsive to ICI therapies (19).

### *H pylori* Infection Decreases the Effectiveness of Cancer Immunotherapies in Pre-Clinical Models

In order to evaluate whether *H pylori* decreases ICIs efficacy, we tested whether *H pylori* reduce the effectiveness of anti-CTLA4 and/or anti-PD-L1 therapy in the MC38 colon adenocarcinoma model. Interestingly, the tumor volumes of *H pylori* infected mice undergoing treatment were significantly larger than those of non-infected mice (19). Furthermore, we tested whether *H pylori* reduces the effectiveness of a cancer vaccine in the B16-OVA melanoma model. Mice subcutaneously injected with B16-OVA melanoma cells were intravenously administered with CD8^+^ T cells specific for OVA and vaccinated with OVA peptide adjuvanted in CpG (70). Remarkably, the tumor volumes of vaccinated infected mice were significantly larger than those of vaccinated non-infected mice, showing that anti-cancer vaccination is less efficient in *H pylori* infected mice (19). Lastly, by using the colon cancer model induces by the administration of azoxymethane (AOM) and DSS, we evaluated the effect of *H pylori* infection on immunotherapy in tumors developing *in situ* (71). Remarkably, the administration of anti-CTLA4 antibody was more efficient to reduce the number of colon tumors in non-infected mice than in infected mice (19). Collectively, we observed that in pre-clinical models, *H pylori* infection reduces the cancer immunotherapy effectiveness.

### *H pylori* Seropositive Non-Small Cell Lung Cancer Patients Demonstrated Reduced Efficacy of Anti-PD1 Immunotherapy

We performed a retrospective study in two independent cohorts of NSCLC patients to evaluate the correlation between *H pylori* infection, diagnosed by serology, and cancer immunotherapy efficacy. Out of 60 NSCLC patients in a French cohort, 18 patients were tested *H pylori* seropositive. Out of 29 NSCLC patients of the second cohort (Canada), 8 patients were tested *H pylori* seropositive. Remarkably, in the French cohort, a decrease of NSCLC patient survival upon anti-PD-1 therapy were associated with *H pylori* seropositivity. *H pylori* seropositive patients demonstrated survival medians of 6.7 months versus 15.4 months for seronegative patients. Additionally, in the second cohort, a decrease of NSCLC patient progression free survival upon anti-PD-1 therapy was observed in *H pylori* seropositive patients compared to seronegative counterparts. Moreover, a numerical difference for overall survival was also observed in the Canadian cohort, since NSCLC patient survival was 21.7 months for seronegative patients compared to 9.3 months for seropositive patients. Collectively, we observed in two independent cohorts of NSCLC patients that *H pylori* seropositivity is associated with lower effectiveness of anti-PD-1 immunotherapy (19). A limitation of our data is that the *H pylori* seropositivity is not synonymous of active *H pylori* infection, therefore prospective studies detecting past and active *H pylori* infection are necessary to confirm these results in cancer patients.

### *H pylori* Infection Blocks Tumor Specific Immune Responses Initiated by Cancer Immunotherapies

The triggering of strong tumor specific immune responses are key for the efficacy of cancer immunotherapies. Remarkably, in the tumors of *H pylori*-infected mice, we detected a decreased number and activation status of tumor-specific T cells. In addition, the vaccine-induced tumor-specific T cells generated in *H pylori* infected mice displayed lower cytotoxic activities than those generated in non-infected mice. These results show that, in *H pylori* infected mice, tumor-specific immune responses primed by vaccination are reduced (19).

The functions of DC and more particularly those implicated in the CD8^+^ T cell priming have been previously been shown to be modulated by the gut microbiota (72, 73). We hypothesized that *H pylori* infection decreases the abilities of DC to prime tumor-specific immune responses. We indeed observed activation defects of DCs in the tumor draining lymph nodes of infected mice. Secondly, an *in vitro* co-culture assay showed that splenic DCs of infected mice do not promote efficient tumor specific T cells proliferation. Collectively, it can be speculated that *H pylori*-induced DC defects (69, 74) are at least partially responsible for the low efficacy of cancer immunotherapies in infected hosts (19).

Remarkably, we detected decrease activation of innate immune responses in infected mice. Indeed, 24 hours post injection of the cancer vaccine, a large decrease of serum inflammatory cytokine levels was detected in infected mice compared to non-infected counterparts. Among other, the level of IFNγ, a cytokine that is critical to sustain anti-tumor responses, was dramatically lower in the serum of infected mice compared to non-infected counterparts. Collectively, since we observed that *H pylori* infection jeopardizes the activation of the innate immune response, we hypothesized that these defects are key in the reduction of efficacy of cancer immunotherapies in infected hosts (19). Consequently, one might postulate that cancer immunotherapies that do not rely on a strong activation of innate immune responses such as CAR-T/TCR-T could be as effective in patients infected or not by *H pylori*.

## Exploring the *H pylori*-Dependent Physiological Mechanism(s) Leading to the Decrease of the Tumor-Specific Immune Responses Initiated by Cancer Immunotherapies

We showed that by dampening the cross-presentation activities of DC and the production of inflammatory cytokines, *H pylori* prevents the triggering of an optimal tumor-specific immune responses. However, we did not identify the *H pylori*-dependent physiological mechanism(s) leading to these immune defects. Studying the physiological mechanisms which lead to *H pylori*-induced hypo-responsiveness to ICIs may allow for the identification of new therapeutic approaches that better promote tumor immune responses and/or the selection of more appropriate therapeutic options for patients with cancer. We will discuss the different *H pylori*-dependent physiological pathways that might lead to decreased efficacy of cancer immunotherapies in *H pylori infected* hosts.

### *H pylori* Infection Modulates the Composition of the Gut Microbiota

The presence of *B. fragilis*, *B. thetaiotaomicron* and *Burkholderiales* in the gut microbiota, described for the first time by Vetizou et al., was shown to be critical for the efficacy of anti-CTLA4 blockade in mice (12). Later, *A. muciniphila, F. prausnitzii*, and *Bifidobacterium* species were also demonstrated to promote responses to immunotherapies (13, 14). More importantly, data collected in humans clearly demonstrate that the gut microbiome also plays critical roles on the responses to anticancer immunotherapies (13, 14, 75) and that antibiotics were shown to be associated with a decrease efficacy of programmed PD-1 blockade therapy (15). Since, it well described that *H pylori* infection modulates the gut microbiota composition (76), we evaluated whether immune-potentiating bacteria such as *B. fragilis*, *B. thetaiotaomicron* and *Burkholderiales, A. muciniphila, F. prausnitzii*, and *Bifidobacterium* are present in the gut microbiota of *H pylori* infected mice. To probe for the role of the gut microbiota the immunosuppression induced by *H plyori*, three experimental protocols were conducted. Firstly, co-housing experiments were conducted. Mice are coprophagic, hence mice reared in the same cage display very similar fecal microbiota composition. Interesting enough, *H pylori* infection is not transmitted when infected mice are reared with non-infected mice (19). Using the B16-OVA melanoma model, we observed that in co-housed mice, the tumor volumes of vaccinated infected mice were larger than those of vaccinated non-infected mice (19). Similarly, anti-CTLA4 therapy was less efficient in co-housed infected mice comparted to non-infected counterparts (19). Next, 16S rRNA gene sequencing was perform to determine the composition of the fecal microbiota. As already described by Kienesberger *et al.* (76), we observed that the composition of the fecal microbiota of non-infected and infected mice differed. Notably, we observed a reduction of *Lachnospiraceae* and *Erysipelotrichaceae* genera, and an increased of the *Bifidobacterium* genus in the fecal microbiota of *H pylori* infected mice compared to non-infected counterparts. Additionally, non-infected mice demonstrated lower alpha diversity as compared to infected mice. Remarkably enough, *H pylori infected* individuals compared to non-infected controls also displayed increased alpha diversity (77). Paradoxically, the high *Bifidobacterium* colonization and high alpha diversity are commonly associated with good responses to cancer immunotherapies in pre-clinical models (72, 78). Lastly, using the B16-OVA melanoma model, we performed fecal transplants. Feces from *H pylori infected* mice were administered to non-infected mice and cancer immunotherapies them performed. We observed that the transplantation of *H pylori* infected feces into non-infected mice does not decrease the efficacy of the cancer vaccination to control the tumor growth. Inversely, *H pylori infected* mice, transplanted with feces of non-infected mice maintained a decreased efficacy of cancer immunotherapies (19), suggesting that FMT will not improve the responses of *H pylori* infected cancer patients to immunotherapies. Although the fecal microbiota of *H pylori* infected host differs from non-infected mice, these differences do not play a major role in the low efficacy of cancer immunotherapies in infected hosts (19). However, our data do not address the notion that *H pylori*-mediated immunosuppression of cancer immunotherapies may rely on the microbiota of the small intestine (79) or stomach (80, 81).

### *H pylori* Infection Modulates Dendritic Cell Activities

It has been described that the gut microbiota promotes the maturation of DCs. The gut microbiota-dependent DC maturation drives potent tumor-specific immune responses and augments the activity of CTLA-4 and/or PD-L1 blockades (12, 78). For exemple, in mice administered with *B. fragilis* it has been observed that the efficacy of anti-CTLA-4 blockade is increased. This B. fragilis-dependent potentiation effect was associated with an induction of Th1 responses in the lymph nodes draining the tumor and with the maturation of DCs in the tumor bed (12). Additionally, DC activation was shown to be sustained by *Bifidobacterium* and associated with the induction of very potent tumor-specific CD8^+^ T cell responses leading to the improvement of the efficacy of PD-L1 blockades (72). Favorable microbiota was also shown to promote the secretion of IL-12 by DCs to recruit, in the tumor beds, CCR9^+^CXCR3^+^CD4^+^ T cells (14). The microbiota has also been shown to modulate the functions of plasmacytoid DCs. Indeed, type I interferon produced by plasmacytoid DCs was shown by modulating the metabolic and epigenomic state of conventional DCs, to increase the priming of T cell responses (82). Importantly, Tanoue K et al. showed that the oral gavage with 11 bacterial strains enhances the therapeutic efficacy of ICIs in a CD8^+^ T cell dependent manner (83). Though the authors did not identify the cellular and molecular mechanisms by which the 11 strains promote systemic anti-tumor responses, plausible hypotheses include the systemic circulation of bacteria, bacterial antigen, bacteria-loaded DCs, metabolites (induced or produced by the 11 strains) and/or cytokines.

In our context, one might hypothesize that products released by *H pylori* may directly and/or indirectly stimulate DCs within the tumor microenvironment or in the tumor-draining lymph node to compromise the priming of tumor-specific CD8^+^ T cells (19).

**Figure 1:**
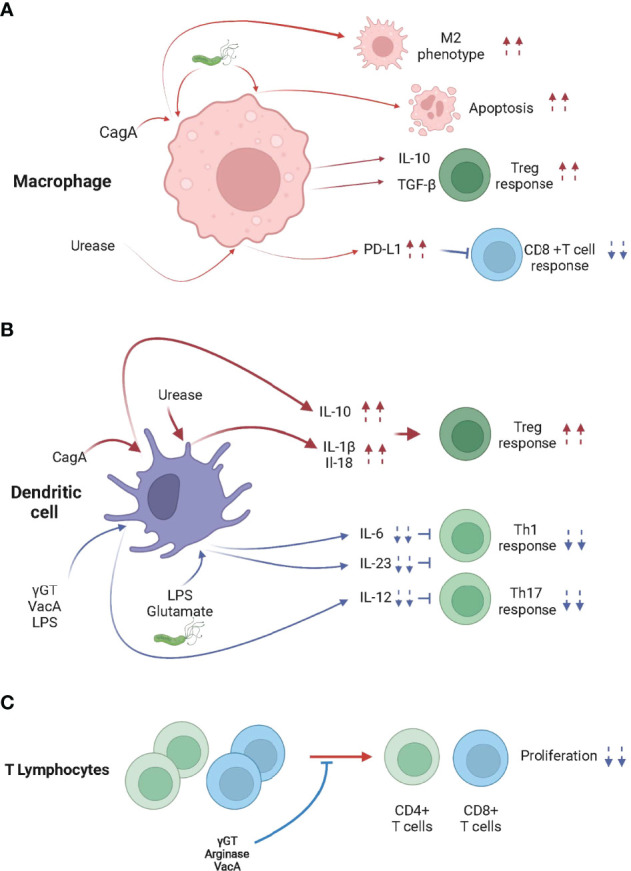
*H pylori* infection modulates the activity of macrophages, dendritic cells and T lymphocytes. **(A)** When in contact with *H pylori*, cytotoxin-associated gene A (CagA) and/or urease, skew macrophages toward an M2 phenotype, which promotes Treg cell differentiation, inhibits CD8^+^ T cell responses and/or promotes apoptosis. **(B)** *H pylori*, CagA, γ-glutamyl transpeptidase (γGT), vacuolating cytotoxin A (VacA), Lipopolysaccharide (LPS), glutamate and urease trigger the differentiation of tolerogenic DCs, leading to the Treg cell-skewed Th response. **(C)** γGT, VacA and Arginase inhibit the proliferation of CD4^+^ and CD8^+^ T cells.

*H pylori* infection has, indeed, a major impact on the function of DCs (**Figure 1A**). *In vivo*, *H pylori* was shown to modulate DCs to induce tolerance rather than immunity to *H pylori* (84, 85) or allergic airway diseases (74, 85). *In vitro*, Kao et al. showed that *H pylori*–pulsed bone marrow derived DCs (BM-DCs) produced significantly lower levels of IL-6 and IL-23 compared with DCs pulsed with *Escherichia coli* or *Acinetobacter lwoffii* (86). In addition, they showed that *H pylori*–pulsed BM-DCs preferentially induced Treg cells rather than Th17 cells (86). Remarkably, *in vitro* and *in vivo* experiments showed that DCs exposed to live or *H pylori* extracts are less capable of processing the antigen (74, 87). In addition, Oertli M et al. showed that *H pylori* mutants lacking γGT or VacA, induced the maturation of BM-DCs more efficiently than wild-type bacteria and showed a reduced ability to inhibit the activation of BM-DCs by LPS of *E. Coli* (88). They also showed that BM-DCs pulsed with γGT or VacA *H pylori* mutants displayed reduced ability to induce Treg cells compared to wild-type *H pylori* (88). These two *H pylori* virulence determinants expressed by all clinical isolate were also shown to be critically involved in the promotion of *H pylori* persistence, and protection against allergic airway diseases through tolerogenic reprogramming of DCs (88). Additionally, CagA was shown to dampen DC maturation in mice (89), and to induce tolerogenicity upon activation of the transcription factor STAT3 and IL-10 secretion in human DCs (90). BM-DCs exposed to *H pylori* extracts produced and secreted large amounts of IL-10, which was dependent on toll-like receptor 2 (TLR2) and Myd88 signaling, but independent on TLR4 (69).

Urease is a secreted and non-secreted protein that has been shown to activate NLRP3 in a TLR2 dependent manner and induce the processing and secretion of the caspase-1-dependent cytokines IL-1β and IL-18 in BM-DCs (91–93). *Caspase-1*ko, *IL-18*ko, *IL-18R*ko, *Nlrp3*ko or *Tlr2*ko mice control *H pylori* infection more effectively than WT animals because they fail to induce Treg cells (85, 93, 94). Importantly, neonatal infection with urease deficient *H pylori* generated low caspase-1 activation, low NLRP-3 expression, low IL-18 secretion and did not efficiently protect from allergen-induced asthma (94). Taken together, urease, in addition to its acidity buffering role, is key in the *H pylori*-induced immune tolerance both locally and systemically.

The binding of *H pylori* LPS to DC-SIGN expressed by DCs resulted in signals which decrease the production of IL-6 and IL-12 and block Th1 cell responses (95, 96). DC-SIGN signaling, by favoring the production of IL-10, a non-inflammatory cytokine, decreases expression of inflammatory cytokines initiated by TLR activation (96). Remarkably, human DCs differentiated from circulating monocytes and infected with *H pylori* also produced in a DC-SIGN, TLR2 and TLR4 dependent manner IL-10.

Glutamate, produced as the result of γGT enzymatic activity, activates glutamate receptors on DCs, inhibiting cAMP signaling and the release of the proinflammatory cytokine IL-6, favoring the induction of a Treg cell response over Th1 and Th17 responses (97). Recently, it was shown that the metabotropic glutamate receptor 4 (GRM4) suppresses antitumor immunity and antagonizes the treatment of ICIs (98).

One may hypothesize that the DC tolerance induced by *H pylori*-derived molecules such as γGT, VacA, urease, LPS and/or glutamate may be involved in the *H pylori*-induced hypo-responsiveness to cancer immunotherapies (**Figure 1A**). The defects in activation of DC in the tumor draining lymph nodes of infected mice in as little as 24 hours post tumor cell engraftment and the lower cross-presentation activity of splenic DCs of *H pylori infected* mice support this hypothesis (19).

### *H pylori* Infection Modulates Macrophage Function

It has been reported that macrophages interact with *H pylori* within the gastric mucosa (99) and that numerous factors released by *H pylori*, such as urease (100), heat shock protein 60 (Hsp60) (101), and/or LPS (102, 103) were shown to activate macrophages. Remarkably, *H pylori* impedes the phagocytic killing capacity of macrophages (104) and may promote macrophage apoptosis (105, 106). It has been shown that macrophages infiltrating the gastric mucosa of *H pylori* infected hosts display the pro-inflammatory (M1) profile (107). Nonetheless, macrophages isolated from the gastric mucosa of *H pylori*–infected mice also demonstrate anti-inflammatory (M2) characteristics such as the activation of the arginase/ornithine decarboxylase metabolic pathway (106, 108). Remarkably, infected patients also show an increase of M2 markers in their gastric mucosa (109). Moreover, studies have associated the production of IL-10 and TGF-β (99) by macrophages, the typical M2 macrophages cytokines, with *H pylori* infection (109). *H pylori* increases the expression of the anti-inflammatory heme oxygenase-1 (HO-1) *enzyme in macrophages* (110). This process requires CagA phosphorylation and the activation of p38 and NF (erythroid-derived 2)-like 2 (NRF-2). The increased activity of HO-1 reduces the pro-inflammatory M1 markers and promotes of the M2 profile of the infected macrophages, leading to defects in the initiation of innate and adaptive immune responses (**Figure 1B**). Because tumor associated macrophages tend to be considered M2-skewed macrophages in the majority of established tumors (111), *H pylori* infection might remotely strengthen the M2 phenotype within tumors and/or tumor draining secondary lymphoid organs, lowering the efficacy of cancer immunotherapies in chronically infected hosts (112).

One may hypothesize that the M2 phenotype induced by *H pylori*-derived molecules, such as CagA, might be involved in the *H pylori*-induced hypo-responsiveness to cancer immunotherapies. The significant decrease of the serum levels of inflammatory cytokines in infected mice day one post cancer vaccination supports this hypothesis (19).

### *H pylori* Infection Modulates T Cell Function

*Multiple virulence factors of H pylori* were shown to directly limit adaptive immune responses by restricting T cell proliferation. This *H pylori*-dependent inhibition of T cell proliferation may be partly responsible for the decreased efficacy of cancer immunotherapies in *H pylori* infected hosts.

*H pylori* arginase, by depleting L-arginine, was shown to inhibit T cell proliferation and reduce the expression of the T cell receptor zeta-chain (113). Similarly, γGT impairs T-cell activation, proliferation and the production of cytokines by specifically depriving glutamine from the extracellular space (114). Endocytosis of the pathogenic factor VacA into activated human primary T cells (115) was also shown to inhibit cell proliferation and the clonal expansion of T cells (116, 117) (**Figure 1C**).

**Figure 2:**
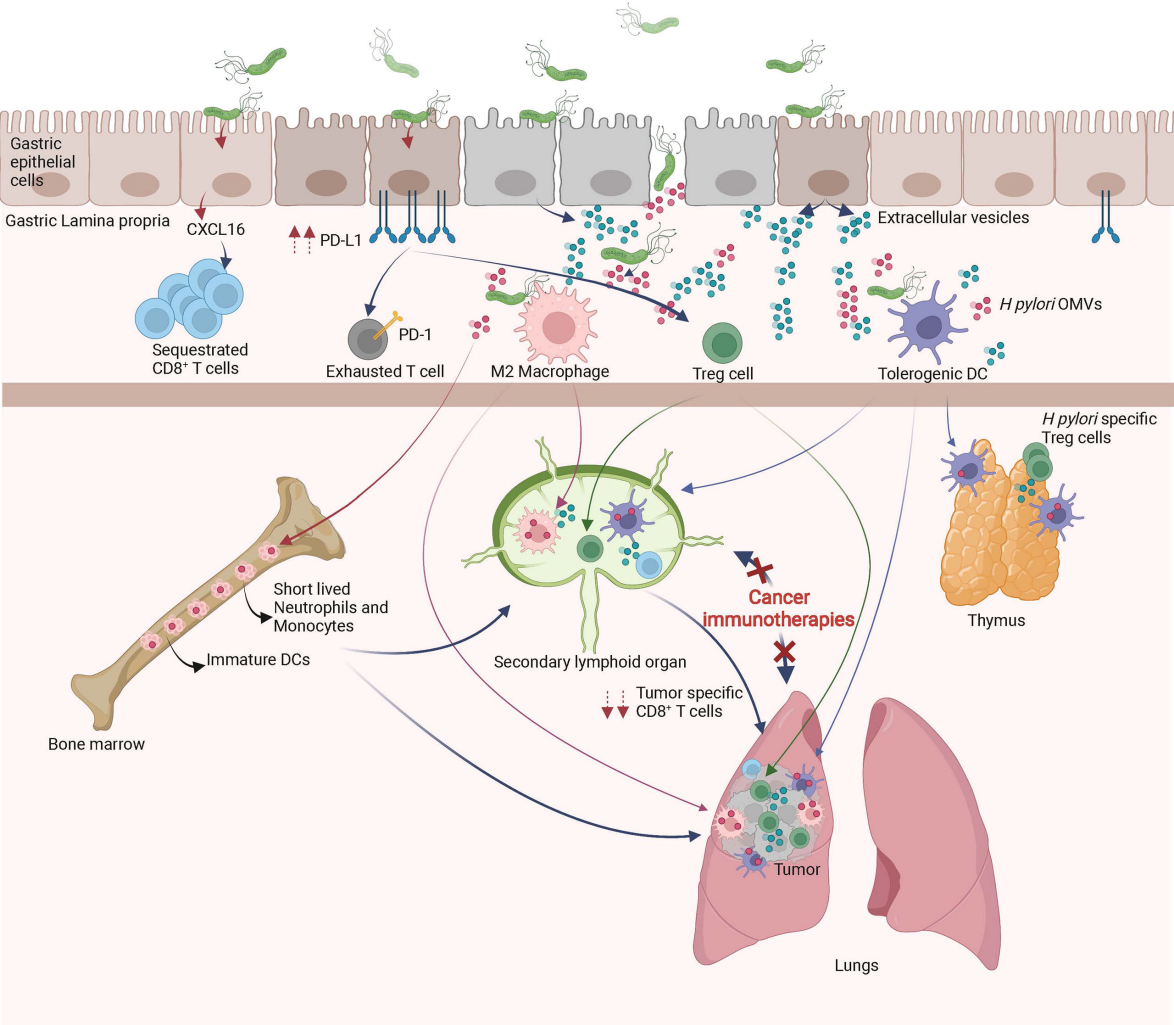
Local and remote effects of *H pylori* infection on host immune functions. In the gastric mucosa, *H pylori* promotes the differentiation of tolerogenic DCs, M2 macrophages, Treg cells, inhibits Th1/Th17 responses and/or sequesters CD8^+^ T cells. The systemic diffusion/migration of extracellular vesicles, outer membrane vesicles (OMVs), Treg cells, tolerogenic DCs and M2 macrophages modulates the function of primary and secondary lymphoid organs, leading to a decrease in tumor specific immune responses triggered by cancer immunotherapies.

In addition to the direct effects of *H pylori* virulence factors on T cell function, *H pylori* was shown to modulate the function, survival and/or homing of T cells by acting on gastric epithelial cells, macrophages and DCs. Recently, Wu J et al. showed that the effect of urease on macrophages leads to the suppression of cytotoxic CD8^+^ T cell responses through the induction of PD-L1 (118) (**Figure 1B**). Similarly, increased expression of PD-L1 in gastric epithelial cells is observed in *H pylori* infected hosts, leading to suppression of IL-2 synthesis and CD4^+^ T cell proliferation (119). In addition, it was shown that the high PD-L1 expression (120) and also the production of transforming growth factor beta (TGFβ) expression (121) preferentially promote the maintenance of Foxp3^+^ Treg cells within the gastric mucosa (**Figure 2**).

As described above, *H pylori* infection induces tolerogenic properties of DCs, macrophages and gastric epithelial cells, all of which lead to Treg cell differentiation. High numbers of Treg cells is beneficial in the maintenance of chronic infection however, is detrimental for the efficacy of cancer immunotherapies. Indeed, *H pylori*-induced Treg cells may seed tumor sites and/or secondary lymphoid organs, weakening the initiation and/or effector phase of anti-cancer immune responses initiated by cancer immunotherapies (**Figure 2**). This hypothesis is supported by previous studies showing that a favorable microbiota in positive responders to CTLA-4 or PD-1 blockade have lower numbers of peripheral blood and tumor Treg cells (122, 123). Importantly, *H pylori*-induced Treg cells have been shown to reduce the strength of the immune responses initiated in the lungs of rodents suffering from experimental asthma (124).

In addition, chronic *H pylori* infection may skew anti-cancer immune responses by decreasing the ability of tumor-specific CD8^+^ T cells to access the tumor bed and/or tumor draining lymph nodes. Indeed, by sustaining chronic immune activation within the gastric mucosa, *H pylori* may attract immune effectors cells continuously in the gastric mucosa, gastric lamina propria and gastric draining lymph nodes. It has been recently shown that *H pylori*–induced matrix metallopeptidase-10 promotes the gastric homing of CD8^+^ T cells through the secretion of CXCL16 (125). One might hypothesize that naïve CD8^+^ T cells or cancer immunotherapy-primed CD8^+^ T cells may preferentially home to the gastric mucosa instead of the tumor draining lymphoid organs or tumor bed, respectively (**Figure 2**). This hypothesis is supported by two recent papers showing that *H pylori* infection modulates schistosome-induced liver fibrosis or mycobacterial infection (126, 127).

### *H pylori* Infection Modulates Natural Killer T Cells Functions

Natural killer T (NKT) cells recognize, within the context of CD1d, glycolipid antigens. Recent studies have demonstrated that gut microbiota modulate the function of systemic and mucosal iNKT cells (128, 129). Remarkably, a study by Ito et al. showed that cholesteryl α-glucosides of *H pylori* are recognized by iNKT in the stomach mucosa. The activation of iNKT cells limits *H pylori* infection by promoting inflammatory responses (130). In addition, it was shown that PI57, a cholesterol-derived lipid from *H pylori*, protects against the development of allergen-induced airway hyperreactivity (131).

NKT cells display strong anti-tumor responses and thus are a major target in the development of cancer immunotherapies. Following treatment with α-Galactocylceramide, a potent activator of iNKT cells, iNKT cells synthetize multiple cytokines modulating the functions of macrophages, neutrophils, NK cells, DCs, T cells, and B cells. Moreover, activated iNKT cells can directly induce cell death in tumor cells. Mice lacking the iNKT cell subset, compared with wild-type mice, respond to CTLA-4 blockade with markedly increased cure rate and overall survival, indicating that iNKT cells might play a role in efficacy of cancer immunotherapy (132).

Therefore, it may be hypothesized that the function of mucosal and/or systemic iNKT cells are modified by *H pylori* cholesteryl α-glucosides, leading to the decrease in efficacy of cancer immunotherapy protocols (19).

### *H pylori* Infection Modulates Gastro-Intestinal Permeability

*H pylori* is known to induce chronic inflammation and directly increase epithelial permeability by redistributing tight junction protein ZO-1 in gastric epithelial cells (20, 133–135) leading to a leaky gut. It has been described that during cancer immunotherapy treatment, the gut barrier is altered (12, 71). Therefore, the leaky gut induced by anti-ICIs and *H pylori* infection may cause an influx of antigens and immunomodulatory molecules produced by *H pylori* and other constituents of the gut microbiota in mucosal and systemic tissues. These microbiota derived-molecules modulate the mucosal and systemic immune responses (20, 71, 136) and may potentially decrease cancer immunotherapy effectiveness in *H pylori infected* hosts (19).

## Is the *H pylori*-Induced Decrease in Efficacy of Cancer Immunotherapies Dependent on the Systemic Diffusion of *H pylori* Gut Microbiota-Derived Molecules?

A very elegant and highly informative experiment performed by the group of A Macpherson clearly demonstrated that bacterial-derived molecules are detectable in peripheral tissues (137), leading to the conclusion that many metabolic activities of our organism rely on gut microbiota-derived molecules. For instance, molecules produced or modified by the gut microbiota, such as short chain fatty acids (SCFAs; acetate, butyrate and propionate), tryptophan pathway metabolites or bile acids have been shown to diffuse freely throughout the gastro-intestinal epithelial cells and modulate the function of our immune system.

### A Role for SCFAs?

SCFAs are the products of the microbial fermentation of nondigestible nutritional fibers. In the human gut, the phylum Firmicutes are the main butyrate producing-bacteria. In particular *Faecalibacterium prausnitzii* and *Clostridium leptum* of the family *Ruminococcaceae*, as well as *Eubacterium rectale* and *Roseburia* spp. of the family *Lachnospiraceae* produce butyrate (138). The SCFA receptors belong the family of G protein-coupled receptors (GPCRs). GPR43, GPR41, GPR109a and GPR164, are expressed by many cells belonging to the gastrointestinal tract and the nervous and immune systems (139). Increased antitumor responses and longer progression-free survival were associated with high fecal SCFA levels, whereas low treatment responses were associated with high systemic SCFA levels (140). Butyrate may inhibit the capacity of DCs to promote tumor-specific T cells, thereby limiting the efficacy of anti-CTLA-4 blockade. Trompette et al. showed that propionate treatment of mice led to modulations of function of hematopoiesis (141). Indeed, the administration of propionate leads to the seeding of the lungs with DCs with impaired ability to promote T helper cell effector functions but with high phagocytic capacity. More specifically, they showed that four days after allergen challenge, the DCs of propionate-treated mice displayed a reduced expression of CD40, PD-L2 and CD86, which is associated with low production of IL-4, IL-5, IL-13, IL-10 and IL-17A by CD4 T cells (141). Taken together, high SCFAs production might account for the observed deficient DC cross presentation activities, low anti-tumor CD8^+^ T cell cytotoxicity and increased Treg cell number in *H pylori* infected mice. However, it was recently shown that fecal SCFA levels are decreased in *H pylori* infected mice compared to the non-infected counterparts, suggesting that CSFAs do not play a role in the reduced efficiency of cancer immunotherapies in *H pylori* infected hosts (142).

### A Role for Tryptophan-Derived Metabolites?

Gut microorganisms such as *Escherichia coli, Lactobacillus reuteri* and *Lactobacillus johnsonii* can metabolize tryptophan into indole-3-acetaldehyde and indole acetic acid (IAA), both of which are aryl hydrocarbon receptor (AhR) agonists. These agonists modulate the functions of many cell types such as epithelial cells, immune cells and astrocytes, both locally and systemically (143, 144). Although recent studies show that tryptophan-derived microbial metabolites suppress anti-tumor immunity by activating AhR in tumor-associated macrophages (145), data are lacking to determine whether tryptophan-derived metabolites are implicated in *H pylori*-induced reduction of cancer immunotherapies (146).

### A Role for Bile Acids?

Primary bile acids are produced by the liver and conjugated to glycine (in humans) or taurine (in mice) transported to the gallbladder and the proximal small intestine, after which they are metabolized by gut microbes to generate secondary bile acids. In intestinal epithelial cells, macrophages among other cell types, microbiome-produced deoxycholic acid and lithocholic acid can bind the GPCR TGR5, as well as the nuclear receptor farnesoid X receptor (FXR) (147, 148). DCs and macrophages both express TGR5 and FXR receptors and secondary bile acids, which have been shown to modulate the anti-cancer immune response, promote anti-inflammatory responses in these cells (149) (150). To our knowledge, no data have been published on the modulation of the production of secondary bile acids in *H pylori* infected hosts. Additional studies are necessary to evaluate whether secondary bile acids play a role in *H pylori*-induced reduction of cancer immunotherapies.

### A Role for Outer Membrane Vesicles or Extracellular Vesicles?

The detection of *H pylori* specific-cell mediated and humoral immune responses in mucosal and systemic compartments is a clear demonstration that *H pylori*-derived antigens can gain access to mucosal and systemic secondary lymphoid organs (20).

Derived from the bacterial outer membrane*, H pylori* secrete outer membrane vesicles (*H pylori*-OMVs). *H pylori*-OMVs carry many of molecules expressed at the surface of the bacteria, such as LPS and peptidoglycan. 77% of the bacterial outer membrane proteins were detected by mass spectrometry analysis in *H pylori*-OMVs. For instance, BabA and SabA adhesins, VacA, CagA, urease subunits, γGT, the protease HtrA, as well as GroEL, catalase are detected in *H pylori*-OMVs (151). Remarkably, *H pylori*-OMVs have been shown to deliver peptidoglycan to the cytosol of gastric epithelial cells, activating in a NOD1-dependent manner NF-KB the production of CXCL-2 and IL-8 (152).

In addition, extracellular-vesicles (EVs) are released from pathogen-infected host cells (EVs). Host-derived EVs like OMVs contribute to the local dissemination of pathogenic components. Pathogenic molecules can be transported between cells *via* EVs and therefore contribute to host-pathogen interactions. EVs may activate and modulate the immune system functions or other cellular signaling pathways. Very interestingly, CagA is present in EVs isolated from the serum of *H pylori* infected patients (153). Therefore it can be hypothesized that CagA derived from *H pylori*-infected gastric epithelial cells can diffuse systemically and gain access to distant organs and tissues such primary or secondary lymphoid organs. Thus, the extra-gastric disorders associated with CagA-positive *H pylori* infection might be induced by the systemic diffusion of CagA-containing exosomes. Although *H pylori*-OMVs are not detectable in the serum of infected individuals at steady state, one may hypothesize that the anti-ICIs-induced gut barrier dysfunctions might induce a systemic influx of *H pylori*-OMVs and CagA^+^-EVs in cancer patients. The *H pylori*-OMVs and CagA^+^-EVs may modulate the function of DCs, macrophages, iNKT cells and T cells, leading to decreased efficacy of cancer immunotherapies (**Figure 2**).

### A Role for Systemic Homing of Mucosal-Derived Myeloid and Lymphoid Cells?

In addition to *H pylori*-OMVs and CagA+-EVs, *H pylori* crossing the epithelial mucosa promote gastric inflammation. In fact, direct contact (including, in some cases, intracellular penetration) between *H pylori*, *H pylori*-derived antigens and stromal cells such as lymphocytes, plasma cells, DCs, macrophages, and granulocytes has been observed in the gastric tissue (154). Occasionally, *H pylori* have also been detected inside blood capillaries, a finding that is in agreement with the transient detection of *H pylori* bacteremia (155). Collectively, these observations show that *H pylori* and *H pylori*-derived antigens are interacting locally with immune cells.

In a very elegant paper, Morton et al. showed that myeloid and lymphoid cells migrate from the periphery to mucosal tissues and migrate back from the gastro-intestinal mucosa to seed peripheral secondary lymphoid organs (156). Therefore, it might be hypothesized that myeloid cells (monocytes and/or DCs and/or macrophages) alongside B and T lymphocytes educated by *H pylori* or *H pylori*-derived antigens within the gastric mucosa migrate to the periphery. Therefore, it can be anticipated that the anti-ICIs-induced gut barrier dysfunctions (12) in *H pylori infected* hosts, mucosal-derived tolerant myeloid and lymphoid cells massively invade the secondary lymphoid organs and tumor bed, leading to decreased efficacy of cancer immunotherapies (19) (**Figure 2**).

### A Role for the Modulation of Primary Lymphoid Organ Functions?

It has been shown that the complexity of the intestinal microbiota strongly correlates with the size of the pool of bone marrow myeloid cells (157). It was also shown that the gut microbiota, *via* TLR ligands, regulates the lifespan of inflammatory monocytes and neutrophils, two myeloid cell populations that are key players in inflammatory responses (158). Therefore, one may hypothesize that in *H pylori infected* hosts, the anti-ICIs-induced gut barrier dysfunctions (12) lead to an influx of *H pylori*-derived antigens such as LPS, urease, VacA, and CagA into the bone marrow. These *H pylori*-derived antigens might modulate the number, lifespan and/or activation status of myeloid cells produced by the bone marrow (**Figure 2**). As a consequence, in *H pylori infected* hosts, the myeloid cells freshly produced by the bone marrow might be deficient in the priming of tumor-specific CD8^+^ T cells following of cancer immunotherapy treatment. The significant decrease of serum levels of inflammatory cytokines of *H pylori infected* mice on day one post cancer vaccination supports this hypothesis (19).

Another hypothesis that could account for *H pylori*-induced hypo-responsiveness to cancer immunotherapies is the massive neonatal thymic production of *H pylori*-specific Treg cells (65). Indeed, it has been shown that a trafficking of gut microbiota-derived antigens to the thymus by DCs occurs during the microbiota colonization in early life. This DC-mediated trafficking of microbiota antigens promotes the differentiation of microbiota-specific Treg cells (159). Therefore, during neonatal *H pylori* infection, the thymus will most probably generate a huge number of *H pylori*-specific Treg cells. Later in life and in the context of *H pylori*-induced and anti-ICIs-induced gut leakiness, the *H pylori*-derived antigens might induce the activation of Treg cells in the tumor draining lymph nodes and/or within the tumor microenvironment and dampen the priming and/or effector phases of cancer immunotherapies (**Figure 2**).

### A Role for the Neonatal Infection?

Upon delivery, neonates leave a sterile environment and are colonized by microorganisms that establish and mold the immune system of the host at both short and long term (128, 160). Depending which microorganism first colonizes the host, the immune system may be heavily impacted. For instance, the neonatal ileum colonization by the segmented filamentous bacteria (SFB) primes a very strong intestinal Th17 response (161). Associated with secretary immunoglobulin A response, Th17 cells control the degree of SFB colonization in young mice as well as other invading pathogens, such as *Citrobacter rodentium* and *Salmonella typhimurium* in adult mice. Very importantly, the influence of SFB on the immune system can have effects on peripheral sites. The host can benefit from SFB colonization; indeed, in predisposed non-obese diabetic mice SFB has been shown to delay the development of diabetes. Inversely, by favoring Th17 cell differentiation, SFB colonization may promote the development of arthritis or multiple sclerosis (162). Another example is the neonatal infection by mouse mammary tumor virus (MMTV). MMTV is produced in the mammary glands of mothers, which allows for viral transmission to neonates during suckling. Very interestingly, the MMTV genome encodes a superantigen that will cause the depletion of subsets of CD4^+^ T cells, creating a hole in the CD4^+^ T cell repertoire for the lifetime of the infected host (163).

In the case of early-life exposure to *H pylori*, it has been shown to induce immune tolerance. This immune tolerance is pivotal to prevent inflammation-induced immunopathology and excessive effector T-cell response to infection, allowing high-level of colonization (164). In addition, early-life exposure to *H pylori* protects from allergic airway disease while the protective effects in mice infected as adults was much weaker (65) Contrastingly, *H pylori* infection of adult mice is associated with higher degree of gastric inflammation and low-level of colonization (164). Collectively, these data suggest that infection with *H pylori* during the neonatal period is a key factor in the *H pylori*-induced local and systemic immune tolerance. Similarly, one may hypothesize that early-life exposure to *H pylori* may be critical in the *H pylori*-mediated immunosuppression of cancer immunotherapies (19).

## Conclusion

More than 30 years of research on *H pylori* has generated amazing insights into the biology of *H pylori*-immune system cross-talks. An array of *H pylori*-derived factors are known to interact with host cells and signaling pathways, leading to chronic immune cell activation in infected hosts. *H pylori-*derived molecules such as VacA, γGT, CagA, urease, arginase, Hsp60, LPS, and/or peptitoglycan generate local and systemic pro- and anti-inflammatory cells and molecules which lead to a general state of hypo-responsiveness. On the one hand, *H pylori*-induced immune tolerance is beneficial to protect individuals from developing severe inflammatory syndrome such as asthma, EoE, IBD and food allergy. However, on the other hand, the immune hypo-responsiveness is most probably the cause of the low efficacy of cancer immunotherapies in *H pylori* seropositive NSCLC patients (19). In addition, as suggested by Shi et al. (165), the relative resistance of gastric cancers to anti-PD1/PD-L1 blockade therapies may also originate from *H pylori*-induced hypo-responsiveness.

Studying the physiological mechanisms which lead to *H pylori*-induced hypo-responsiveness to ICIs may allow for the identification of new therapeutic approaches that better promote tumor immune responses and/or the selection of more appropriate therapeutic options for patients with cancer.

## Author Contributions

PO, LV, BMM and DV wrote the manuscript. All authors contributed to the article and approved the submitted version.

## Funding

Open access funding provided by University of Lausanne.

## Conflict of Interest

The authors declare that the research was conducted in the absence of any commercial or financial relationships that could be construed as a potential conflict of interest.

## Publisher’s Note

All claims expressed in this article are solely those of the authors and do not necessarily represent those of their affiliated organizations, or those of the publisher, the editors and the reviewers. Any product that may be evaluated in this article, or claim that may be made by its manufacturer, is not guaranteed or endorsed by the publisher.
