# Erratum: The efficacy of cancer immunotherapies is compromised by *Helicobacter pylori* infection

**DOI:** 10.3389/fimmu.2023.1183107

**Published:** 2023-03-21

**Authors:** Frontiers Production Office

**Affiliations:** Frontiers Media SA, Lausanne, Switzerland

**Keywords:** *Helicobacter pylori*, cancer, immunotherapy, personalized medicine, gut microbiota, immune checkpoint inhibitors

An omission to the funding section of the original article was made in error. The following sentence has been added: “Open access funding provided by University of Lausanne”.

The original version of this article has been updated.

